# Successful resection of a large adenoma of the descending duodenum by endoscopic submucosal dissection

**DOI:** 10.1055/a-2491-1530

**Published:** 2025-01-21

**Authors:** Xiaolin Chen, Hongna Lu, Qide Zhang, Liangshun Zhang, Ting Weng, Minying Zhu, Feng Xu

**Affiliations:** 1 Department of Gastroenterology, The Affiliated LiHuili Hospital of Ningbo University, Ningbo, China; 2 Digestive Endoscopy Center, Affiliated Hospital of Nanjing University of Chinese Medicine, Jiangsu Province Hospital of Chinese Medicine, Nanjing, China; 3 Department of Anesthesiology, The Affiliated LiHuili Hospital of Ningbo University, Ningbo, China

A 59-year-old man was admitted to our hospital with a diagnosis of hypopharyngeal carcinoma. During gastroscopy, a mass was identified in the descending part of the duodenum, and biopsy results indicated the presence of low grade intraepithelial neoplasia. An enhanced computed tomography of the upper abdomen demonstrated that the wall of the descending part of the duodenum was markedly thickened, exhibiting a local mass-like convexity into the lumen, which was markedly and homogeneously enhanced ( Fig. 1 ), with mesenteric arterial blood supply. Additionally, the local lumen of the duodenum was narrowed, and the surrounding fat space was clear. Following the exclusion of contraindications, a decision was taken to proceed with endoscopic submucosal dissection (ESD).

**Fig. 1:**
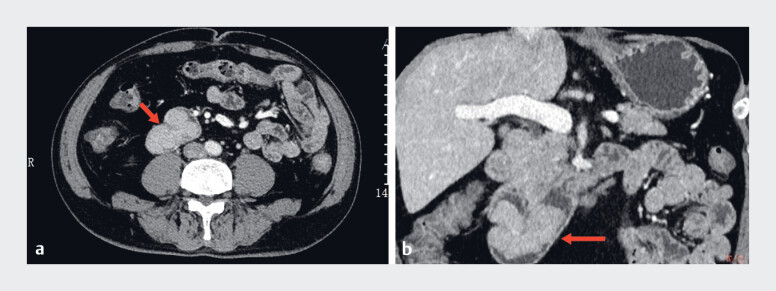
Preoperative computed tomography (CT) scan of the upper abdomen. **a** Transverse CT of the descending duodenal tumor (red arrow). **b** Coronal CT of the descending duodenal tumor (red arrow).

Prior to incision in the form of a “big C,” epinephrine was injected submucosally ( Fig. 2 **a–c** ). Dissection was then conducted in a layer-by-layer manner, during which white adhesions were observed ( Fig. 2 **d, e** ). The entire lesion was excised. The wound was treated with electric coagulation using hot biopsy forceps before being sutured with clips and dental floss ( Fig. 2 **f, g** ). A three-chamber gastric feeding tube was placed ( Video 1 ). The lesion measured 9.6 × 5.0 × 1.5 cm in size ( Fig. 2 **h** ). Histological examination confirmed complete resection of a tubular villous adenoma with glandular low grade intraepithelial neoplasia.

**Fig. 2:**
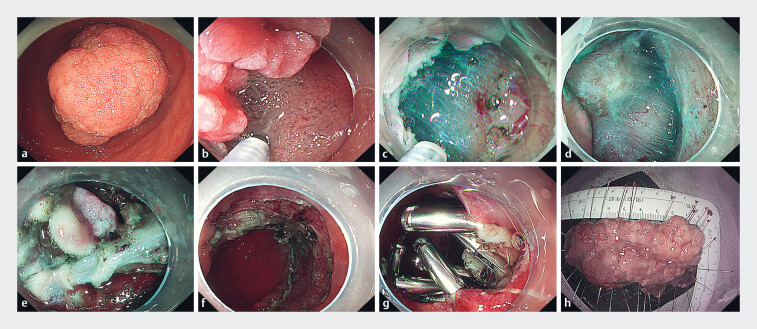
Endoscopic submucosal dissection of a large adenoma of the descending duodenum. **a** The large adenoma of the descending duodenum. **b** Submucosal injection of epinephrine melphalan. **c** Mucosal incision in the form of a “big C.” **d** Dissection of the submucosa, layer by layer. **e** White adhesions under the mucosa on the anal side. **f** Wound after complete removal of the lesion. **g** Wound suturing using clips and dental floss. **h** The lesion measured 9.6 × 5.0 × 1.5 cm in size.

Successful resection of a large adenoma of the descending duodenum by endoscopic submucosal dissection.Video 1

The three-chamber gastric feeding tube was removed on the sixth postoperative day, and a semi-liquid diet was started, with gradual transition to a normal diet. No complications, such as bleeding or perforation, were observed during this time. A follow-up gastroscopy 1 month after surgery revealed the presence of a linear, reddened post-ESD scar in the descending duodenum ( Fig. 3 ). Additionally, the intestinal lumen was observed to be smooth and free of stenosis.

**Fig. 3:**
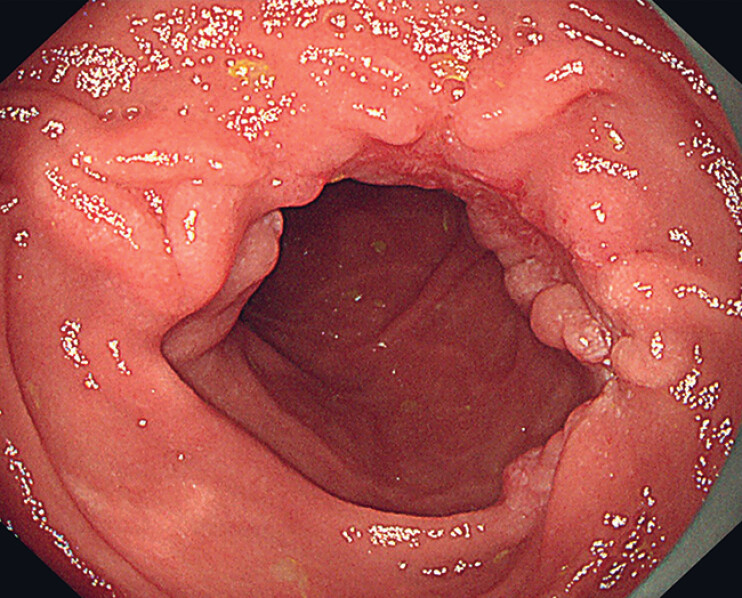
Repeat gastroscopy performed 1 month after surgery demonstrated a linear, reddened scar in the descending duodenum, with a patent intestinal lumen and no evidence of stenosis.

Given the rarity of duodenal tumors, there is a paucity of literature on large duodenal adenomas. The distinctive anatomical characteristics of the duodenum, including a small lumen and a C-shaped cavity, present challenges in performing ESD 1 . This report details a rare case of descending duodenal macroadenoma that was successfully and completely resected by ESD.

Endoscopy_UCTN_Code_TTT_1AO_2AG_3AD

## Notes

Endoscopy E-Videos https://eref.thieme.de/e-videos
*E-Videos* is an open access online section of the journal *Endoscopy* , reporting on interesting cases and new techniques in gastroenterological endoscopy. All papers include a high-quality video and are published with a Creative Commons CC-BY license. Endoscopy E-Videos qualify for HINARI discounts and waivers and eligibility is automatically checked during the submission process. We grant 100% waivers to articles whose corresponding authors are based in Group A countries and 50% waivers to those who are based in Group B countries as classified by Research4Life (see: https://www.research4life.org/access/eligibility/ ). This section has its own submission website at https://mc.manuscriptcentral.com/e-videos .
